# Egg Consumption and Incidence of Heart Failure: A Meta-Analysis of Prospective Cohort Studies

**DOI:** 10.3389/fnut.2017.00010

**Published:** 2017-03-27

**Authors:** Owais Khawaja, Hemindermeet Singh, Faraz Luni, Ameer Kabour, Syed S. Ali, Mohammed Taleb, Hafeezuddin Ahmed, John Michael Gaziano, Luc Djoussé

**Affiliations:** ^1^Department of Cardiology, Mercy St. Vincent Medical Center, Toledo, OH, USA; ^2^Division of Aging, Brigham and Women’s Hospital, Harvard Medical School, Boston, MA, USA; ^3^Massachusetts Veterans Epidemiology and Research Information Center (MAVERIC), Boston Veterans Affairs Healthcare System, Boston, MA, USA; ^4^Preventive Medicine, Brigham and Women’s Hospital, Harvard Medical School, Boston, MA, USA; ^5^Geriatric Research, Education, and Clinical Center (GRECC), Boston Veterans Affairs Healthcare System, Boston, MA, USA

**Keywords:** eggs, heart failure, nutrition, epidemiology, diet

## Abstract

**Introduction:**

Heart failure (HF) remains a major health problem affecting 5.7 million adults in USA. Data on the association of egg consumption with incident HF have been inconsistent. We, therefore, conducted this meta-analysis of prospective cohort studies to assess the relation of egg consumption with incident HF in the general population.

**Methods:**

Using extensive online search, we conducted a meta-analysis of new onset HF following exposure to egg consumption. A random effects model was used and between studies heterogeneity was estimated with *I*^2^. Publication bias was assessed graphically using a funnel plot. All analyses were performed with Comprehensive Meta-Analysis (version 2.2.064).

**Results:**

We identified four prospective cohorts for a total of 105,999 subjects and 5,059 cases of new onset HF. When comparing the highest (≥1/day) to the lowest category of egg consumption, pooled relative risk of HF was 1.25 (95% confidence interval = 1.12–1.39; *p* = 0.00). There was no evidence for heterogeneity (*I*^2^ = 0%) nor publication bias. On sensitivity analysis, stratification by gender differences, follow-up duration, and region where study was conducted did not alter the main conclusion.

**Conclusion:**

Our meta-analysis suggests an elevated risk of incident HF with frequent egg consumption.

## Introduction

Heart failure (HF) is highly prevalent in clinical practice affecting Americans ≥20 years of age (1). The incidence of HF approaches 10 per 1,000 population after 65 years of age and remains high around 20% at 80 years of age (2). Although, survival after HF diagnosis has improved over the years, death rate continues to remain high at ~50% within 5 years (3). HF has a major economic impact with total projected cost by year 2030 being $69.7 billion, an increase from estimated $30.7 billion for the year 2012 (4).

The traditional risk factors for HF include coronary heart disease (CHD), hypertension (HTN), diabetes mellitus (DM), cigarette smoking, obesity, dietary sodium intake, and valvular heart disease (5). Overall, CHD has been shown to account for about 62% (68% in men and 56% in women) of all HF cases. Eggs are an important source of phosphatidylcholine in human diet (6). In addition, they are an important source of various beneficial minerals and nutrients like vitamin A, vitamin D, calcium, xanthophyll, and folate (7). Dietary choline has been associated with increased trimethylamine-N-oxide (TMAO) production, which in turn may be an important predictor of CHD (8).

Although data on the association of egg consumption with CHD or stroke are inconsistent in the general population (9–11), an increased risk for CHD, stroke, and mortality has been noted among those with DM (12–14). In a recent meta-analysis, frequent egg consumption was also shown to be associated with incident DM (15). However, data on the association of egg consumption with incident HF have been conflicting (16–18).

Identification of simple and inexpensive yet effective strategies to help prevent incident HF can be of paramount importance. This can help reduce not only the overall morbidity and mortality as well as the healthcare costs related to HF. We, therefore, conducted this meta-analysis of population bases prospective cohort studies to examine the association of egg consumption with incident HF in humans.

## Materials and Methods

### Search Strategy

We conducted a search in PubMed, Cochrane library, and Google Scholar up to May 2016 for studies that reported the association of egg consumption with incident HF. We used the following keywords for our search: eggs, nutrition, and HF. The search was performed for studies in English language and was limited to human subjects. When an abstract from a meeting and a full article referred to the same report, only the full report was included in the analysis. In case of multiple reports from the same study, we used the most complete and/or recently reported data. References of the retrieved articles and review articles were also manually screened for eligible studies.

### Inclusion and Exclusion Criteria

We included population-based prospective cohort studies reporting on the association of egg consumption and incident HF. Only the studies comparing event rates between two or more groups with complete information available were included.

### Data Extraction

Data for each study were abstracted by an investigator (Owais Khawaja) and were confirmed by a second investigator (Hemindermeet Singh). Each author used the same template to extract first author’s name, year of publication, country where study was conducted, population characteristics (mean age and range, gender proportion, number of DM, sample size), study design (cohort vs case–control), mean follow-up, categories of egg consumption, person-time of follow-up within each category of egg consumption for cohort, statistical method used to obtain effect size (logistic regression vs Cox proportional hazard model), covariates adjusted for, and relative risk (RR) with 95% confidence interval (CI).

Each author used the same template to extract first author’s name, year of publication, region of study, population characteristics, mean follow-up, categories of egg consumption, statistical method used (Cox proportional hazard model), covariates adjusted for (age, education, physical activity, body mass index, smoking, alcohol consumption, history of DM, atrial fibrillation, HTN, etc.), and RR with 95% CI. In case of a disagreement, the discrepancies were resolved by group discussion between the reviewers.

### Statistical Analysis

The meta-analysis was performed by computing RR using random effects model. RR for incident HF was calculated along with the 95% CIs. Between studies, heterogeneity was analyzed by means of *I*^2^, which describes the percentage of the variability in effect estimate across studies that is due to heterogeneity rather than sampling error (chance). Publication bias was assessed graphically using a funnel plot. We also conducted sensitivity analysis to evaluate the impact of excluding studies with (a) only men/women, (b) <20 years follow-up duration, and (c) US/Non-US studies on the pooled RR. All analyses were performed with Comprehensive Meta-Analysis (version 2.2.064).

## Results

**Figure 1:**
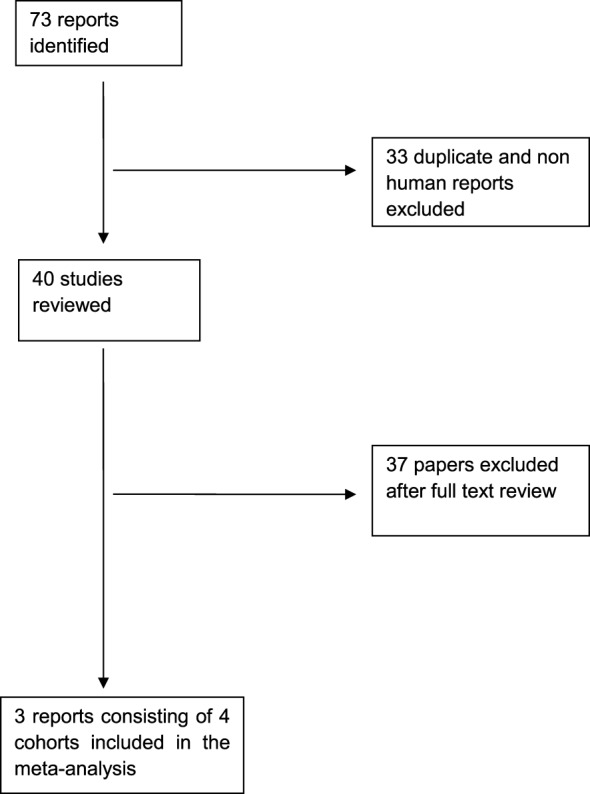
**Search strategy results**.

Overall, we found 73 reports on the primary search and excluded 33 studies because of duplication or non-human subjects. From the remaining 40 reports, we included 3 reports consisting of four cohorts after full text review. All included studies were population-based prospective cohort studies. Figure 1 summarizes the results of literature search along with excluded studies. Basic characteristics of these studies are shown in Table 1.

**Table 1:** **Baseline characteristics of study included in meta-analysis**.

First author, publication year, country	Study name	Study size	Age range (years)	Follow-up period (years)	Dietary assessment	Exposure categories	No of subjects (lowest vs highest category)	No of cases (lowest vs highest category)
Djousse (men), 2008, USA	Physician’s health study	21,275	53–57	20.4	Semi quantitative FFQ	Six groups: <1/week, 1/week, 2–4/week, 5–6/week, 1/day, and 2+/day	4,527 vs 264	206 vs 25
Nettleton (men/women), 2008, USA	Atherosclerosis risk in communities	15,143	54–57	13.3	Semi quantitative FFQ	Nine groups: ≤1/month to ≥6/day. Analyzed as per 1 SD difference in dietary pattern score or per 1 daily serving difference in food group intake	13,013 vs 1,140	1,140 total (differential data not available)
Larson (men), 2015, Sweden	Cohort of Swedish men	37,766	59–61	13.0	Semi quantitative FFQ	Four groups: 0–3/month, 1–2/week, 3–6/week, and 1+/day	17,635 vs 1,007	724 vs 72
Larson (women), 2015, Sweden	Swedish mammography cohort	32,805	61–63	13.0	Semi quantitative FFQ	Four groups: 0–3/month, 1–2/week, 3–6/week, and 1+/day	14,756 vs 682	560 vs 37

*FFQ, food frequency questionnaire*.

**Figure 2:**
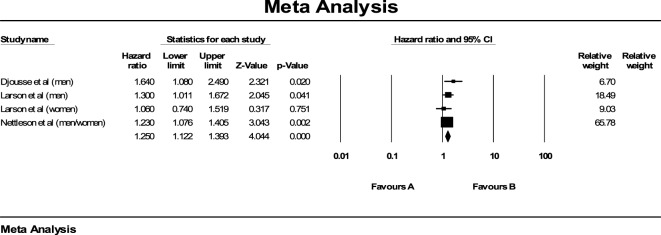
**Pooled relative risk for egg consumption and incident heart failure across prospective cohort studies**.

Three population-based prospective cohort studies with four distinct cohorts were included in this meta-analysis [Djousse (men) (16), Nettleton (men/women) (17), Larsson (men) (18), and Larsson (women) (18)]. For our analysis, we only included those in the highest and the lowest category of egg consumption from each of these cohort studies. Overall, these prospective cohort studies had a total of 106,989 participants with 49,931 (47%) in the lowest and 3,093 (3%) in the highest category (≥1/day) of egg consumption. Larsson (men) (18) had the most number of participants, while Nettleton (men/women) (17) had the least number of participants. Overall, there were 2,764 cases of incident HF. On comparing the highest to the lowest category of egg consumption, the pooled RR for incident HF was 1.25 (95% CI: 1.12, 1.39; Figure 2). There was no evidence for heterogeneity (*I*^2^ = 0%).

**Figure 3:**
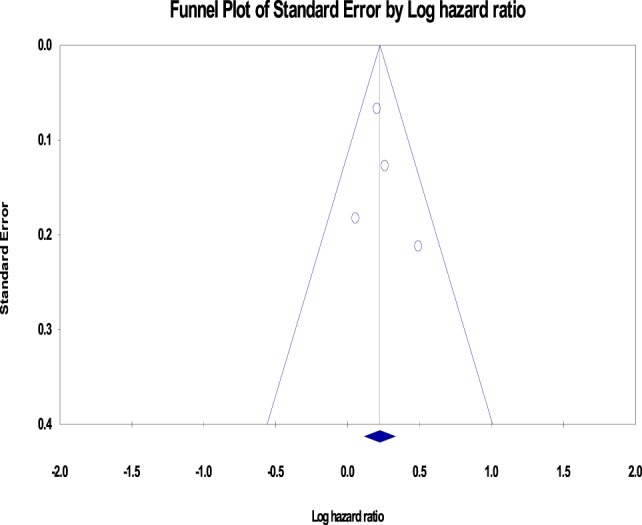
**Funnel plot for pooled analysis for cohort studies**. There is no evidence of publication bias seen.

Sensitivity analysis (stratification by excluding studies with men/women, <20 years of follow-up duration, US/Non-US studies) did not alter the main conclusion (Table 2). There was no evidence of publication bias on funnel plot analysis (Figure 3) as supported by the Egger’s test (*p* = 0.68).

**Table 2:** **Sensitivity analysis**.

Included studies	Relative risk (95% confidence interval)	*p* Value
Excluding studies with women vs all included prospective cohort studies	1.38 (1.12, 1.71) vs 1.25 (1.12, 1.39)	0.414
Excluding studies with men vs all included prospective cohort studies	1.06 (0.74, 1.52) vs 1.25 (1.12, 1.39)	0.389
Excluding studies with <20 years of follow-up vs all included prospective cohort studies	1.64 (1.08, 2.49) vs 1.25 (1.12, 1.39)	0.217
Excluding non-US studies vs all included prospective cohort studies	1.32 (1.04, 1.69) vs 1.25 (1.12, 1.39)	0.687
Excluding US studies vs all included prospective cohort studies	1.22 (0.99, 1.49) vs 1.25 (1.12, 1.39)	0.205

## Discussion

Based on the findings of this meta-analysis, frequent egg consumption was associated with a higher risk of incident HF. In addition, results of sensitivity analysis did not alter the main conclusion.

Data from our study are somewhat consistent with prior studies. Longitudinal data from various population-based studies have shown a steady increase in HF risk secondary to CHD and DM (5, 19, 20). Data on the association of egg consumption with CVD (cardiovascular disease) outcomes have been inconsistent. Shin et al. (12) did not demonstrate that elevated risk for CHD, stroke, or mortality with egg consumption. However, those who ate 1+ egg per day were 42% more likely to develop DM and among those with DM frequent egg consumption was associated with increased CVD comorbidity (HR: 1.69; 95% CI: 1.09–2.62). Similarly, Rong et al. (10) in their analysis demonstrated a higher risk of CHD (RR: 1.54; 95% CI: 1.14–2.09) among people with diabetes. Recently, Djousse et al. (15) in a meta-analysis of prospective cohort studies demonstrated a modestly positive association between frequent egg consumption and incident DM. This was, however, limited to 4+ eggs/week and mostly restricted to US studies. Our analysis did not reveal regional variation with respect to outcome.

Hypertension has been shown to be a more important cause of HF in women, while CHD which has been shown to be the more important cause of HF in men (21). Also, HF with preserved left ventricular ejection is the more common type of HF seen in women. However, we did not have enough number of studies or specific information on the type of HF to make any definitive conclusions. This will need to be further explored in additional studies.

Eggs consumption has been associated with increased production of TMAO (8). Wang et al. (22) in an animal study demonstrated TMAO dietary supplementation of mice to be associated with upregulation of multiple macrophage scavenger receptors linked to atherosclerosis. Tang et al. (23) in their study demonstrated elevated TMAO levels to be associated with an increased risk of major adverse cardiovascular events even after adjustment for traditional risk factors (*p* < 0.001). Senthong et al. (24) in their prospective cohort study of 353 stable CHD patients demonstrated fasting plasma TMAO levels to be an independent predictor of a high atherosclerotic burden. Mafune et al. (25) in their cross-sectional study of 227 patients who underwent cardiovascular surgery demonstrated a significantly increased number of infarcted coronary arteries among those with elevated TMAO levels.

Trøseid et al. (26) in their prospective analysis of 155 patients with chronic HF demonstrated that elevated TMAO levels among patients with advanced HF symptoms and those with ischemic etiology in addition to reduced transplant-free survival over a 5.2-year follow-up (hazard ratio 2.24, 95% CI 1.28–3.92, *p* = 0.005). However, the association of egg consumption with CHD has been inconsistent in general population (9–11), while an increased risk for CHD has been observed among diabetics (12–14). This raises the concern for confounding by dietary patterns. However, due to inadequate available information on relevant dietary factors or dietary patterns in specific subgroups we were unable to further elucidate this issue. Therefore, further studies accounting for comprehensive dietary patterns in addition to consumption of eggs will be needed to explore the underlying biological mechanisms.

Our systematic review has several strengths. All studies included were prospective in nature. The large sample size and prolonged duration of follow-up improved the statistical power to detect smaller effect size. Outcome in the current study was validly ascertained, thereby minimizing misclassification of outcome. On the other hand, our analysis has some possible limitations. Owing to the observational nature of studies, one cannot entirely exclude unmeasured or residual confounding. Also, self-reported egg consumption using FFQ might have introduced exposure misclassification. However, since FFQ was obtained prior to incident HF, such misclassification is more likely to be non-differential. We were unable to account for the various cooking methods of eggs and additives which could be another variation factor. We could not isolate egg consumption from food products containing eggs, which can be a potential bias. We were unable to further look into the composition of eggs and their nutritional quality, which may vary according to the species and the breed of the laying hen. Moreover, we were not able to further classify the type of HF.

Overall, this meta-analysis of population-based prospective cohort studies suggests an elevated risk of HF with frequent egg consumption in humans. Further studies are warranted to explore the underlying biological mechanisms.

## Author Contributions

OK came up with the idea. OK and HS did data collection and analysis. OK, HS, FL, and HA wrote initial version. AK, SA, MT, HA, JG, and LD provided critical input.

## Conflict of Interest Statement

LD is a recipient of an investigator initiated grant from American Egg Board. The reviewer MD and handling editor declared their shared affiliation, and the handling editor states that the process nevertheless met the standards of a fair and objective review.
